# Coarse-Graining ddCOSMO through an Interface between Tinker and the ddX Library

**DOI:** 10.1021/acs.jpcb.2c04579

**Published:** 2022-10-20

**Authors:** Michele Nottoli, Aleksandr Mikhalev, Benjamin Stamm, Filippo Lipparini

**Affiliations:** † Dipartimento di Chimica e Chimica Industriale, Università di Pisa, Via G. Moruzzi 13, 56124Pisa, Italy; ‡ Department of Mathematics, RWTH Aachen University, Schinkelstr. 2, 52062Aachen, Germany

## Abstract

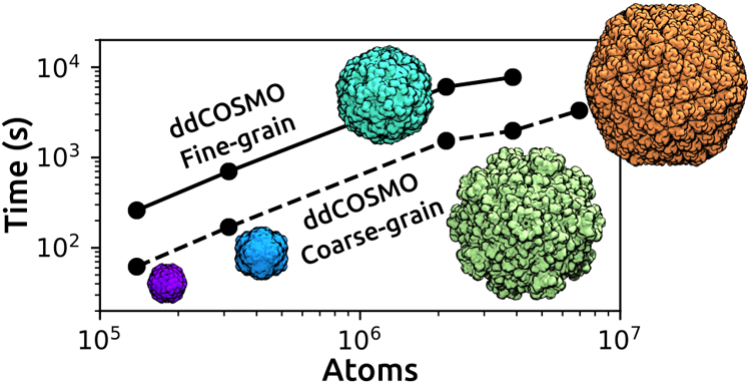

The domain decomposition conductor-like screening model is an efficient way to compute the solvation energy of solutes within a polarizable continuum medium in a linear scaling computational time. Despite its efficiency, the application to very large systems is still challenging. A possibility to further accelerate the algorithm is resorting to coarse-graining strategies. In this paper we present a preliminary interface between the molecular dynamics package Tinker and the ddX library. The interface was used to test a united atom coarse-graining strategy that allowed us to push ddCOSMO to its limits by computing solvation energies on systems with up to 7 million atoms. We first present benchmarks to find an optimal discretization, and then, we discuss the performance and results obtained with fine- and coarse-grained solvation energy calculations.

## Introduction

Polarizable continuum solvation models (PCSM) are well established techniques used to model in a cheap, yet effective way the effects of the environment,^1−6^ let it be a solvent or even more complex matrices, on the molecular properties of the solute, by introducing a polarization term in the solute’s Hamiltonian that includes self-consistently mutual polarization effects. In PCSM, the solute is accommodated in a cavity surrounded by a uniform, infinite dielectric, which is polarized by the solute’s charge distribution. There are many possible definitions for the molecular cavity, which is an empirical construct, as it is not associated with a quantum mechanical molecular observable. The most commonly used definitions are the van der Waals (VdW) cavity,^7−9^ the solvent-accessible surface (SAS),^10−12^ and the solvent-excluded surface (SES), also referred to as Connolly’s surface.^13−15^ The first two cavities are geometrically simple, as they are both the union of interlocking spheres, with radii chosen, respectively, as (scaled) van der Waals radii or van der Waals radii augmented by the radius of a probe that represents the solvent. VdW cavities are commonly used in quantum mechanical calculations, where the solute is a small- to medium-sized molecule. Their use for large solutes can be problematic, as such cavities can have nonphysical holes and crevices, making the SAS a preferred choice. The SES is mathematically a much more complicated construct. There are several algorithms used to construct it,^16−21^ but unfortunatly its application makes analytical energy derivatives particularly hard to formulate. Nevertheless, it is the most commonly used surface to compute solvation-free energies in biophysics.

In standard implementations, the solvent polarization is obtained by solving an integral equation defined at the boundary of the molecular cavity. This is commonly done using dense linear-algebra techniques, that require  flops, where *N* is the number of discretization elements, which in the best possible case is proportional to the surface exposed to the solvent. In standard applications, where the solute is treated using an accurate quantum-mechanical technique, this is overall a small contribution to the total computational effort required to carry out the calculation, which is largely dominated by the cost of solving the quantum mechanical equations. However, if one uses a cheaper semiempirical model or even a multiscale QM/MM approach, the size of treatable systems can become very large, making  operations an insurmountable bottleneck. The computational complexity of PCSM stems from their polarizable nature, that is, they add a genuine many-body contribution to the energy that requires the solution to a linear system of equations whose size is proportional to the global size of the embedded system. To overcome such a bottleneck, formulations that achieve a linear scaling computational cost with respect to the size of the system are mandatory. At the same time, if one wants to use such a model to compute molecular structure and properties, it is paramount that the formulation maintains the numerical accuracy required to compute analytical derivatives, which include not only geometrical gradients, but also, for example, contributions of the environment to the molecular Hamiltonian and response functions.^22^

The domain decomposition algorithm for the conductor-like screening model (ddCOSMO), introduced in 2013 by Cancès, Maday, and Stamm, enjoys all of such properties.^23−25^ The ddCOSMO implementation of the conductor-like screening model^4,26^ naturally enjoys linear scaling properties as the COSMO polarization equations obtained in the dd paradigm are sparse, making the calculation of matrix-vector products possible in  flops and with  memory requirements. In the past decade, ddCOSMO has been implemented in the context of quantum chemistry,^27,28^ polarizable molecular dynamics (MD),^29^ QM/MM, and polarizable QM/MM.^30^ Geometrical gradients, as well as second-order electric properties and excitation energies have been implemented for both QM methods such as Hartree–Fock and density functional theory, as well as semiempirical models. It has been used to perform polarizable MD simulations of small peptides in water^29^ and recently has been coupled in a fully self-consistent way to a polarizable QM/MM strategy based on density functional theory and the AMOEBA polarizable force field.^31^ In all these examples, we have tested ddCOSMO for systems of hundreds and even up to a few tens of thousands atoms. While such systems are large in the context of QM or even QM/MM calculations, they are still quite small when compared to the systems studied in classical simulations, such as large proteins or even entire viruses.^32,33^ Furthermore, even on such large, but not extremely large, systems, ddCOSMO can still be rather expensive, especially when the solution of the ddCOSMO equations is required many times, for example, to compute the self-consistent AMOEBA/ddCOSMO polarization.^34^

In the last years, we have worked on an open-source, state-of-the-art implementation of ddCOSMO, which, together with the domain decomposition implementation of the polarizable continuum model^35−37^ and of the linearized Poisson–Boltzmann model,^38,39^ has been recently released as part of the ddX library.^40^ In this contribution, we have interfaced the ddX library with the Tinker classical MD package.^41^ We push the limits of polarizable continuum solvation by presenting benchmark calculations done with ddCOSMO on systems made by up to 7 million atoms. Furthermore, we present a ddCOSMO implementation for a coarse-grained SAS cavity, which is obtained by using a united-atom topology. Such a cavity has been originally proposed by Barone et al.^42^ in the context of the polarizable continuum model and is made by the union of interlocking spheres, each centered at a heavy atom, with the hydrogen atoms being contained in the spheres around the heavy atoms they are bonded to. We demonstrate that the new implementation, which uses the fast multipole method to compute the solute’s potential, needed to build the right-hand side (RHS) of the ddCOSMO equations, allows one to perform calculations on extremely large systems, even on moderate hardware, paving the way for a polarizable continuum model that bridges the gap between models used in quantum chemistry and biophysics. We also discuss the choice of the numerical parameters that control the ddCOSMO discretization to achieve a desired precision on absolute and relative energies.

The rest of this paper is organized as follows. In the next section, we first present the theory of ddCOSMO and how the united atom coarse-graining strategy is formulated, and then we briefly illustrate the interface between Tinker and ddX. In the Results and Discussion, we first present the benchmarks used to determine suitable discretization parameters, and finally, we present a comparison of the fine- and coarse-grained models on systems of up to 7 million atoms.

## ddCOSMO for a United Atoms Cavity

In COSMO,^4,26^ the solute is accommodated in a cavity in a conductor medium, characterized by an infinite dielectric permittivity, the density of the solute polarizes the environment, and in turn, the polarization interacts with the solute’s density, giving rise to an electrostatic contribution to the solvation energy. The domain decomposition strategy allows one to rewrite the COSMO problem as a collection of local problems, each of them coupled only with the neighboring local problems. In this way, after the discretization, the problem is characterized by a sparse linear system, which can be solved with linear scaling computational complexity and memory requirements.^43^

The only requirement posed by the ddCOSMO method on the cavity definition is that it must be the union of spheres and that it must enclose the solute’s density. From a formal point of view, the second requirement means that the support of the solute’s density must be strictly contained in the cavity, which is not an issue for MM solutes. In all the previous works, the cavity definition is based on a one-to-one correspondence between atoms and spheres, however this is not the only possibility. For example, the number of spheres could be reduced and the radii of the spheres could be enlarged, so that the cavity is simplified or in other words coarse-grained, while still enclosing the solute’s density. Reducing the number of spheres in turn reduces the computational cost associated with assembling the right-hand side and solving the linear system, thus allowing the description of even larger systems.

The cavity coarse-graining requires particular care: the set of rules used to define the radii and positions of the spheres must be differentiable with respect to the atoms’ positions in order to retain the differentiability of the energy, and hence a definition of the forces. In the remaining part of this section, we present the theory of ddCOSMO for a cavity described using the united atom (UATM) strategy, which is one of the most straightforward, yet differentiable, possible strategies. The definition of such a cavity is described in detail in ref (42).

**Figure 1:**
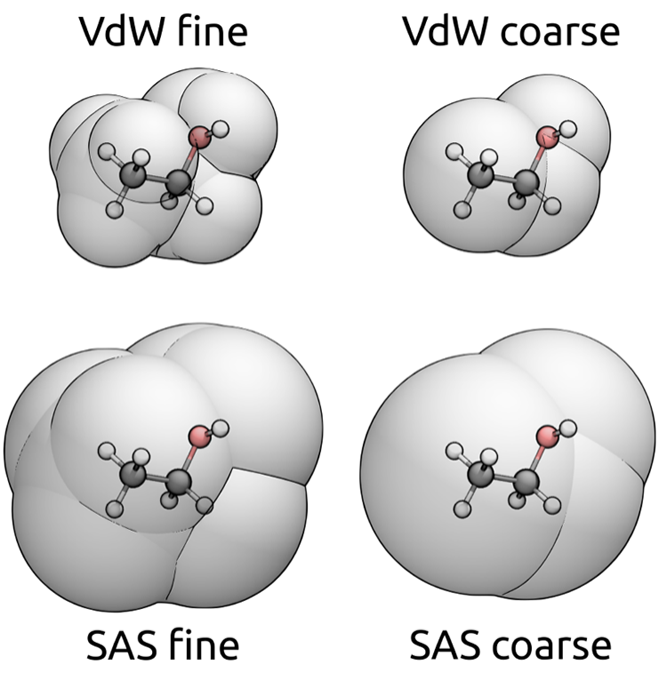
Exampe representation of the four possible surfaces coming from the VdW and SAS definitions combined with the use of the UATM strategy. The radius used for the solvent in the SAS definition correspond to water (1.4 Å).

Let us consider a solute, with its charge distribution ρ, and let  be a molecular cavity that accommodates it. We assume that Ω is the union of *N*_sph_ interlocking spheres Ω_*i*_, as it is the case for van der Waals (VdW) or SAS cavities, and these two kinds of cavities, combined with the coarse- or fine-grain strategies, lead to the four different surfaces that are shown as an example in Figure 1. We also assume that ρ is a distribution of point charges, as it is typical in classical molecular mechanics force fields. The generalization to more advanced charge distributions, including quantum mechanical ones, induced point dipoles, and distributed multipoles, can be found in the relevant literature.^27,29^ However, in this contribution, we no longer assume that each atom is endowed with a sphere, that is, we consider the possibility of having solute’s charges that are not in the center of a sphere.

The ddCOSMO algorithm is an efficient numerical realization of COSMO, that computes a numerical solution to the following partial differential equation (PDE)
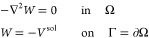
1where *V*^sol^ is the electrostatic potential of the solute computed in vacuo and *W* is the reaction potential. In ddCOSMO, eq 1 is replaced by an equivalent set of coupled differential equations, one for each sphere Ω_*i*_, in the spirit of Schwarz’s domain decomposition method. Let Γ_*i*_ = ∂Ω_*i*_ and let  and  be the portions of Γ_*i*_ that are exposed to the solvent (i.e., ) or buried inside the cavity, respectively. Let also  be the list of spheres Ω_*j*_, *j* ≠ *i* that intersect Ω_*i*_ and let  be the number of intersecting spheres at a specific point *r* ∈ Γ_*i*_. The ddCOSMO coupled integral equations read, for each sphere Ω_*i*_
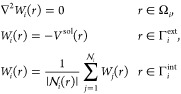
2From eq 2 it is apparent that each equation is only coupled to the equations at neighboring spheres, making the set sparse. It is possible to recast the ddCOSMO equations in a more compact form by introducing a few auxiliary quantities. Let
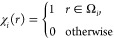
3be the characteristic function of the *i*-th sphere, and let
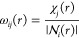
4Using such quantities, we can further define the characteristic function of the external surface Γ as
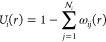
5By using the quantities defined in eqs 4 and 5, the ddCOSMO equations become, for each sphere Ω_*i*_,
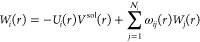
6As each *W*_*i*_ function is harmonic, the ddCOSMO equations can be rewritten as a set of coupled integral equations by introducing, for each sphere, a local apparent surface charge σ_*i*_ such that
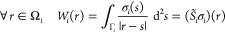
7and
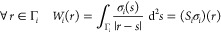
8where we have introduced the single layer potential  and single layer operator *S*.^44^ Note that the formal difference lies in the point *r* where the expression is evaluated. The ddCOSMO set of coupled integral equations becomes, thus
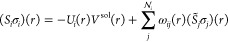
9The ddCOSMO equations are then discretized by expanding the local ASC into a truncated set of real-valued spherical harmonics  up to angular momentum :
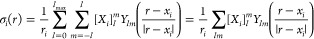
10where *x*_*i*_ is the center of the *i*-th sphere, and we have introduced a short-hand notation for the double sum in . In this way, we can introduce a vector ***X***, which is the collection, for each sphere, of the coefficients of the linear combination of spherical harmonics. The solute’s potential, which constitutes the right-hand side for the ddCOSMO equations, is discretized as
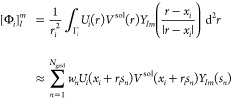
11where we have introduced the Lebedev–Laikov quadrature^45^ (weights and points *w*_*n*_, *s*_*n*_) to compute the surface integral, and *N*_grid_ is the number of Lebedev points on a sphere. Furthermore, to achieve a smooth dependence of the energy with respect to the positions of the spheres (and, therefore, of the atoms), the characteristic function is smoothed based on a regularization parameter η > 0. All the details can be found in ref (24). After discretizing the single layer operators *S* and , one gets a sparse linear set of equations that we write, for brevity, as
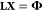
12where **L** is the ddCOSMO matrix and we have collected all the discretized ASCs and right-hand sides in the vectors **X** and **Φ**. The ddCOSMO equations can be efficiently solved by using an iterative solver. In our implementation, we use Jacobi Iterations accelerated with Pulay’s Direct Inversion in the Iterative Subspace (JI/DIIS).^46^ The cost of solving the linear equation is , thanks to the sparsity of the ddCOSMO matrix **L**.

Once the ddCOSMO linear system has been solved, it is possible to compute the electrostatic contribution to the solvation energy as
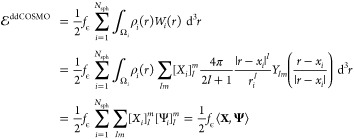
13where *f*_ϵ_ is an empirical scaling factors that accounts for the dielectric nature of the solvent.

The definition of the **L** matrix only depends on the geometry of the cavity, that is, the centers and radii of the interlocking spheres, and is not affected explicitly by the number and positions of the solute’s atoms. In other words, whether an all-atoms or a coarse-grained cavity is used, the structure and size of the matrix is modified but the equations that are used to define it remain the same. On the contrary, **Φ** and **Ψ** depend explicitly on the solute’s charge distribution and its relation with the spheres composing the cavity and are computed using different expressions. In the remaining part of this section we consider the case of a UATM cavity, by first explaining how this coarse-graining strategy work and then by providing the expressions for **Φ** and **Ψ**.

**Figure 2:**
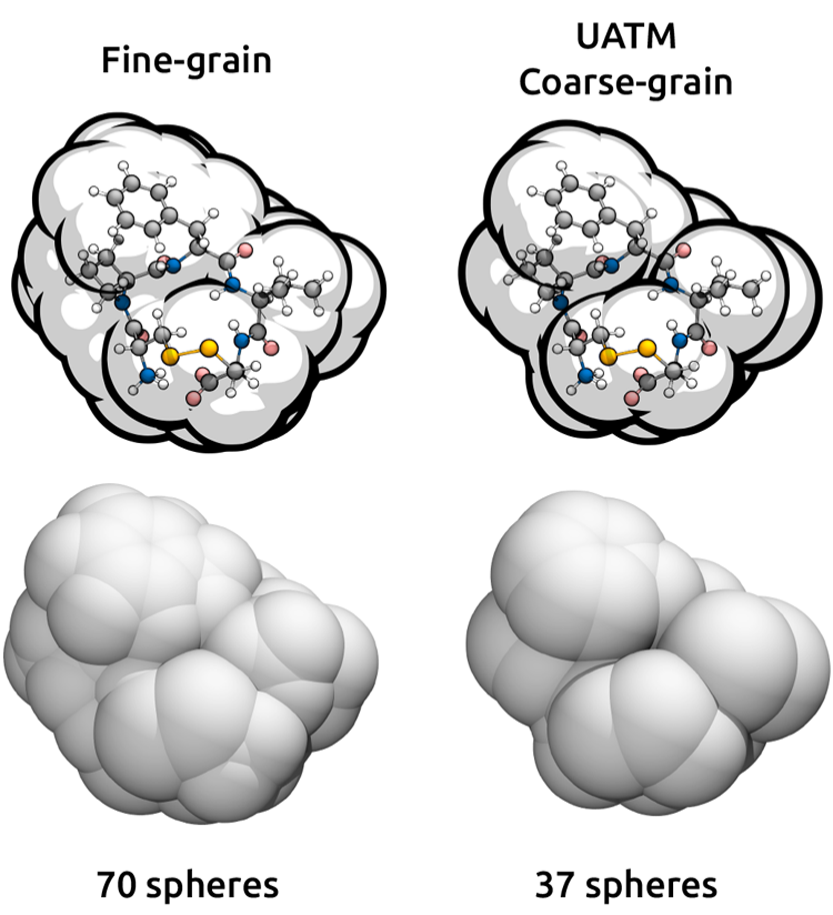
Example of a fine-grained SAS cavity and of a coarse-grained UATM SAS cavity for the same small peptide, PDB code 2p7r (see Table 1).

In the UATM approach, the topology of the system is analyzed and all the spheres that correspond to hydrogens bonded to heavy atoms are removed. At the same time, the radii of the spheres corresponding to the heavy atoms are increased depending on the number of removed bonded hydrogens. The outcome is that the large spheres placed on the heavy atoms contain both the heavy atoms and the hydrogens linked to them. An example of two cavities, a SAS fine-grained cavity and a SAS UATM coarse-grained cavity, are reported in Figure 2, whereas simpler cavities for a VdW surface are shown in Figure 1. The precise rules used for computing the radii are cumbersome as depend on the atomic number and on the hybridization of the heavy atoms, for this reason we refer to the work of Barone et al. for their definitions.^42^

In our implementation, we decided to not only coarse-grain the cavity definition, but to coarse-grain also the multipolar distribution according to the same rules. In other words, the partial charges associated with hydrogens linked to heavy atoms are moved to the heavy atoms themselves using a multipolar translation.

This additional coarse-graining results in the multipoles being at the center of the spheres composing the cavity, and a one-to-one correspondence between multipoles and spheres. In this way there is a large advantage concerning the implementation: the same ddX interface, provided that can handle multipoles of higher order, can handle in the same exact way a fine- and a coarse-grain case, without the need of even knowing that a coarse-grain calculation is being performed.

During the development, we tested also a different strategy in which the multipoles are not coarse-grained, in such a way there is a few-to-one correspondence between multipoles and spheres, and the multipoles are not necessarily at the centers of the spheres. Using either one or the other strategy results in differences in the computation of **Ψ** and **Φ**. For what concerns the first, the coarse-graining of the multipoles results only in a difference in the implementation, from a mathematical point of view, performing a translation and then integrating is equivalent to evaluating the integral in eq 13 with off-centered multipoles. Moreover, also the computational cost is similar, as the operations required in the translation of the multipoles are also required in the computation of **Ψ** with off-centered multipoles. For what concerns **Φ**, the differences are more significant. First, representing the potential of a collection of multipoles with a single multipole obtained through M2M translations is an approximation - and in particular, the same used by the FMM method. Provided that the expansion order is high enough, the approximation is accurate. In practice, we observed that with a maximum angular momentum equal to  the effect on the final results is negligible. Second, there could be an effect on the computational cost of evaluating the potential: both the strategies coarse-grain the cavity, and hence, the number of target points is the same. The strategy which coarse-grains the multipoles has fewer sources, but despite this, it is not necessarily cheaper as the coarse-grained multipoles have a higher angular momentum, and hence a more complicated kernel for the evaluation of the potential. In practice, we observed that the cost of computing Φ using the two strategies is similar due to some cancellation of costs: lower angular momentum and more sources against higher angular momentum and fewer sources.

With this preface on the coarse-graining, the theory required is composed of two parts: a way to perform the multipolar translations, and an expression for **Φ** and **Ψ** for higher order multipolar distributions.

The multipolar translations are performed using a part of the fast multipole method (FMM) machinery,^47^ namely the M2M operator. This operator, in the basis of real spherical harmonics, is represented with a square matrix of size , with  being the selected maximum angular momentum. In the FMM implementation available in ddX,^48^ we apply a rotation-based technique to lower the computational complexity of the operation. The multipoles at the source are rotated such that the positions of the source and of the target are aligned to the OZ axis. Then an M2M operation along the OZ axis is performed, and finally the inverse rotation is applied. The expression of an M2M matrix associated with a translation along the vector ***v*** can be written as
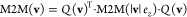
14
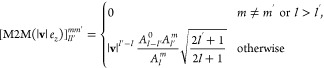
15In this expression, *Q*(***v***) is the rotation matrix that aligns the ***v*** vector with the *e*_*z*_ axis, and .

Once the translated multipolar distribution is assembled, it is possible to compute **Ψ** as
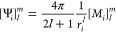
16where ***M*** is the collection of the higher order multipolar distributions in real spherical harmonics, and  is an element of the multipole placed on the *i*-th sphere. For what concerns **Φ**, the first step is assembling the electrostatic potential of the solute’s density at the exposed grid points, and then, expression 11 is used to assemble the desired quantity. For a multipolar distribution in real spherical harmonics, the expression for the electrostatic potential, evaluated in ***x***, reads
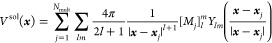
17where ***x***_*j*_ is the position of the *j*-th multipole and *N*_mult_ is the number of multipoles. Assembling the solute’s potential, if done naively, requires  operations, and is therefore, in principle, quadratically scaling with respect to the size of the system. In the current implementation, this bottleneck is removed by using the FMM,^47^ that has been implemented in the ddX library and is part of the publicly available code. The FMM library already accepts multipoles of any order as input, so for further technical details about the computation of the electric potential, we refer to reference^48^ where we present the ddX-FMM implementation. We conclude this section with a remark. The implementation of the coarse-graining scheme could be easily extended to QM densities, as the machinery to compute the **Φ** and **Ψ** quantities for QM solutes is completely general and makes no assumption on the position of the atoms with respect to the centers of the spheres.^28,35^ Note, however, that given the size of treatable full QM solutes, a coarse-grained approach is computationally not very attractive.

### Tinker–ddCOSMO Interface

The UATM coarse-graining scheme was implemented in Tinker 8 together with an interface to the ddX library.^40,41^ Since the ddX library accepts as input multipoles of arbitrary order, the support for advanced force fields is already partially present; however, polarizable force fields, such as AMOEBA, require to account for the mutual polarization between the solute and the continuum and hence require additional steps that have not been implemented yet. At the moment, the Tinker–ddX interface can be used to compute the solvation energy in the case of a nonpolarizable force field, in a fine-grained case and in a coarse-grained case, the latter based on the UATM definition. In this section, we provide technical details about the implementation together with a general overview of the interface for the computation of the solvation energy.

The ddX library is compiled as a shared object that can be linked to the Tinker executables so that it is possible to use the ddX functionalities easily. All the data handling is entirely done in Tinker, which has to keep track of the workspaces, constants, RHSs, and solutions needed and produced by the ddX library.

**Figure 3:**
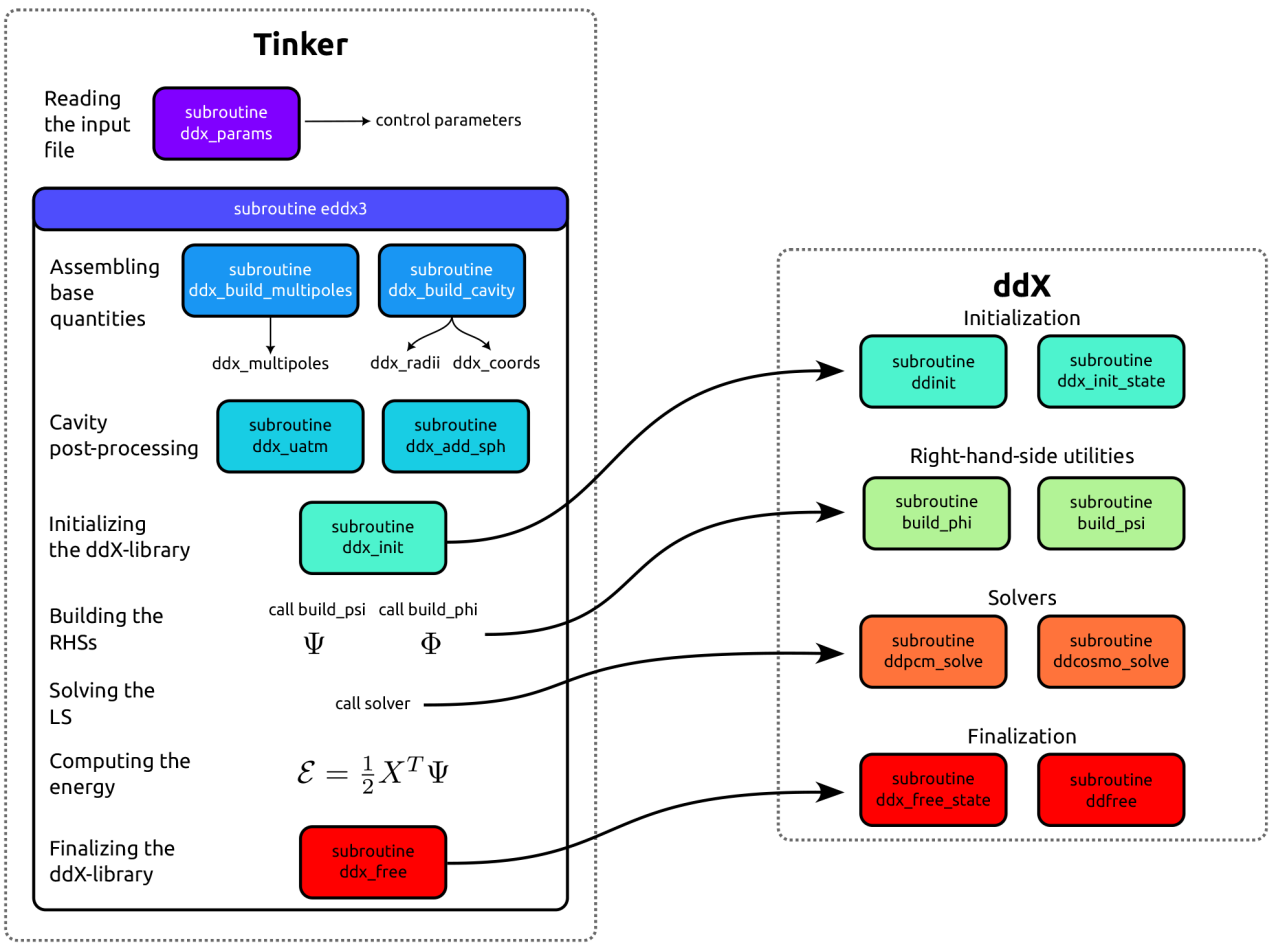
Simplified scheme of the interface between Tinker and ddX. Most of the data handling is omitted for clarity, however, all the subroutines involved are represented with colored boxes.

Figure 3 presents a simplified scheme of the interface, the figure shows the routines exposed by ddX, which have to be called during a continuum solvation calculation and the routines which have been added to Tinker to make the data handling, the logic operation, and the coarse-graining possible.

First, we added a new routine (ddx_parameters) that parses the Tinker input file (.key) for control keywords directly aimed at the ddX library: in this way it is possible to set the model, , *N*_grid_, ϵ, the convergence threshold, the number of OpenMP threads, the use of the FMM, and the maximum angular momentum for the FMM, as well as the coarse-graining method.

Then, the rest of the steps are done within the routine eddx3, which is the top level driver for the computation of the solvation energy. Such a routine also takes care of the initialization of the ddX library. As the various quantities that need to be set before calling the ddX workers depend on the geometry of the system, it has to be done externally, also in the perspective of doing molecular dynamics simulations, that would require to repeat the initialization at every simulation step. In ddX two different routines perform the initialization ddinit allocates temporary workspaces and precomputes the constants used by the method, ddx_init_state allocates space for the RHSs, solutions and all the relevant quantities needed for postprocessing. The latter is a separate routine, so that if needed, more ddCOSMO problems corresponding to different densities can be solved at the same time, while using a common pool of constants and parameters. ddx_init precomputes the scaling factors for the spherical harmonics and the FMM transformations, computes the Lebedev points and weights on the unit sphere and evaluates the spherical harmonics at them, constructs the cluster tree used by the FMM method through recursive intertial bisection,^48^ constructs the neighbor list to be used by ddCOSMO, computes the position of the Lebedev points for all the spheres, and finally, evaluates the functions *U*_*i*_ and ω_*ij*_ at them.

In eddx3, first, various basic quantities such as the cavity definition in terms of radii and centers of the spheres, and the multipoles in real spherical harmonics, are computed. Then, additional processing of the molecular cavity is performed. For example, additional spheres can be appended to the cavity, or the coarse-graining can be applied. The UATM implementation uses the information available in Tinker about the connectivity and about the atom types to decide which atoms are hydrogens bonded to heavy atoms. Then it uses the rules for the cavity together with the M2M translation to modify the three arrays ddx_multipoles, ddx_radii, and ddx_coords. In this M2M translation, we truncate the multipolar distributions at the same value , which is used for the ddCOSMO discretization. It is important to note that the coarse-graining is completely contained in this call, making it possible to implement different coarse-graining schemes without having to modify the remaining part of the implementation.

Once the cavity is available, it is possible to initialize the ddX library, by calling the appropriate routines which allocate the required memory and precompute the constants. The computation of the vectors **Φ** and **Ψ** for multipolar distributions is left to ddX, and then **Φ** is used to solve the linear system **L*****X*** = −**Φ**. Finally, once the solution ***X*** is known, the energy can be computed and the library can be finalized by calling the appropriate subroutines for the deallocation of the memory. In Figure 3 we also reported a solver for ddPCM, which is out of the scope of the present contribution but, at the same time, is implemented in the ddX library and completely shares the interface with ddCOSMO, so it is usable without further modifications.

## Results and Discussion

In this section, we present numerical results that explore the accuracy of the calculation with respect to the choice of the ddCOSMO discretization parameters, and benchmark calculations on a variety of large systems composed of whole viral capsides. The accuracy tests have been performed on a set of smaller structures. The complete list of the used structures is reported in Table 1.

**Table 1:** Details about the Systems Used for Benchmarks on Timings and Energies, the Systems Ranging from 2p7r to 7v7e Have Also Been Used for the FMM and *l*_max_ Bencharks^a^

PDB code	name	ref	N atoms
2p7r	cyclic pentapeptide	(49)	70
1etn	enterotoxin	(50)	143
1du9	scorpion toxin	(51)	381
1gzz	growth factor	(52)	944
1d3w	ferredoxin	(53)	2051
1qgt	hepatitis B (capsid unit)	(54)	9062
1ju2	hydroxynitrile lyase	(55)	20288
7v7e	SARS-CoV-2 spike		47896
			

1stm	satellite panicum mosaic virus	(56)	138408
3n7x	*Penaeus stylirostris* densovirus	(57)	312660
1ohf	*Nudaurelia capensis* omega virus	(58)	2141700
3jay	nuclear polyhedrosis virus	(59)	3863940
1uf2	rice dwarf virus	(60)	6958380

a Top block: smaller systems used also for the FMM and  benchmarks; bottom block: larger systems only used for benchmarks on timings and energies.

The starting point for the calculations are input files for Tinker. To assemble those we used the program pdb2xyz, which is provided by the Tinker package and allows generating a Tinker xyz file from a PDB file and a force field, this tool also protonates the structure according to the valences prescribed by the force field. For generating all the input structures, we used the Amber99 force field.^61^ For the whole viral capsides, the PDB files contain only the structure of the repeating unit, so we used the transformation matrices contained in the PDB header to reconstruct the whole structures.

All the calculations have been performed using the following parameters for ddCOSMO: (i) a convergence threshold of 10^–6^; (ii) a dielectric permittivity ϵ = 80; (iii) an internal switching region η = 0.1. We systematically use a SAS cavity, assembled either using the UATM scheme or directly from the fine-grained list of atoms. In both cases, the effective radius of the solvent (1.4 Å for water) is added to the VdW radius^8^ (or UATM radius) of the atom.

All the calculations have been performed on a single cluster node equipped with 2 AMD EPYC 7282 16-Core @ 2.30 GHz CPUs, for a total of 32 cores, together with 512 GB of RAM memory. Both Tinker and ddX were compiled using the Intel ifort compiler and linked against the Intel MKL libraries.

For the calculations, we used our fork of commit 056b5da of the release branch of TinkerTools/tinker on GitHub, and commit 935a471 of the branch mnottoli/tinker of ACoM-Computational-Mathematics/ddX on GitHub.

### Accuracy of the FMM

In ddCOSMO, the FMM is used to accelerate the computation of the RHS, such that it is done in a linear scaling time. This step is controlled by two parameters, namely the maximum angular momentum of the multipolar (*p*_*m*_) and local (*p*_*l*_) distributions: in the following discussion we set *p*_*m*_ = *p*_*l*_ = *p*_max_. For each test structure, we performed both a fine-grained and a coarse-grained calculation, setting *p*_max_ to the values 2, 4, 6, 8, and 10 and a reference value of 20. For each calculation, we set  and *N*_grid_ = 110.

**Figure 4:**
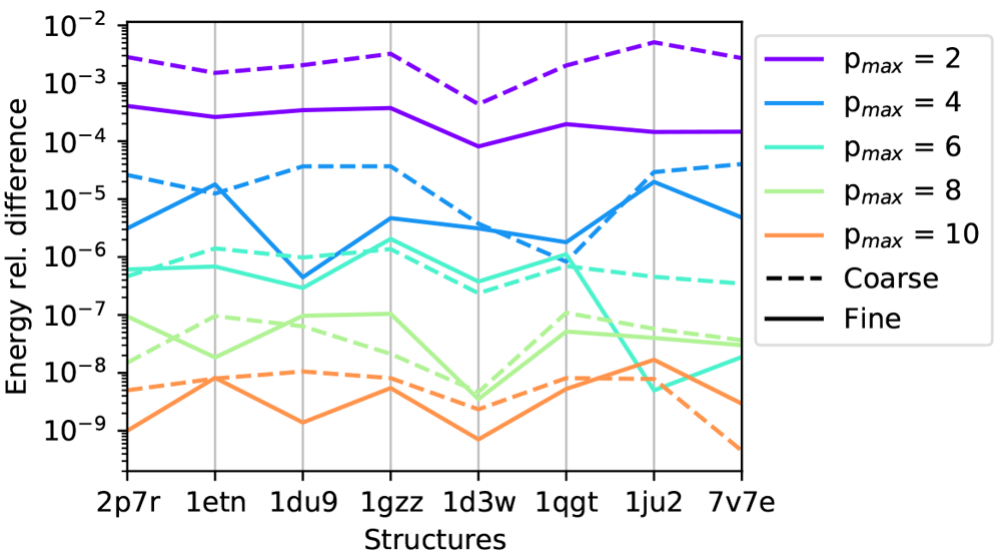
Relative error on the energies computed for the different test structures, using the fine- and coarse-grain implementations, for various accuracies of the FMM. The different colors report various values of *p*_max_.

Figure 4 reports the relative difference on the energy computed with respect to the reference value *p*_max_ = 20, for each system and for the varying control parameters. From this analysis we evidence two main conclusions. First, since in the coarse-graining step we truncate the multipolar distributions at , we need *p*_max_ of at least the same value to avoid a loss of information about the multipolar distribution. When *p*_max_ is equal to 6 the relative difference obtained with a coarse- and a fine-grained model is similar. Second, for what concerns the general accuracy, we note that even with a low value of *p*_max_, we already achieve a relative difference below 1%. The differences are consistent with those found in ref (48), where the FMM implementation available in ddX is used to compute both the electrostatic potential required by the sphere–sphere interactions present in ddPCM and by the RHS as for ddCOSMO.

### Accuracy of the Discretization

As a second preliminary benchmark, we looked for a value of  that provides results with an accuracy of about 1% with respect to a fully converged calculation. To do this, for each test structure, we performed fine- and coarse-grained calculations, setting  to the values 2, 4, 6, 8, 10, 12, and 14 and to a reference value of 20. Each set of fine- and coarse-grained calculations uses as a reference the corresponding fine- or coarse-grained calculation done with . In the fine-grained case, we used a fixed *p*_max_ = 4 for all values of , whereas in the coarse-grained case, we used  to avoid the truncation of the input multipolar distributions. In all the cases, *N*_grid_ was set to a large value of 2030, which is required to properly integrate the spherical harmonics with .

**Figure 5:**
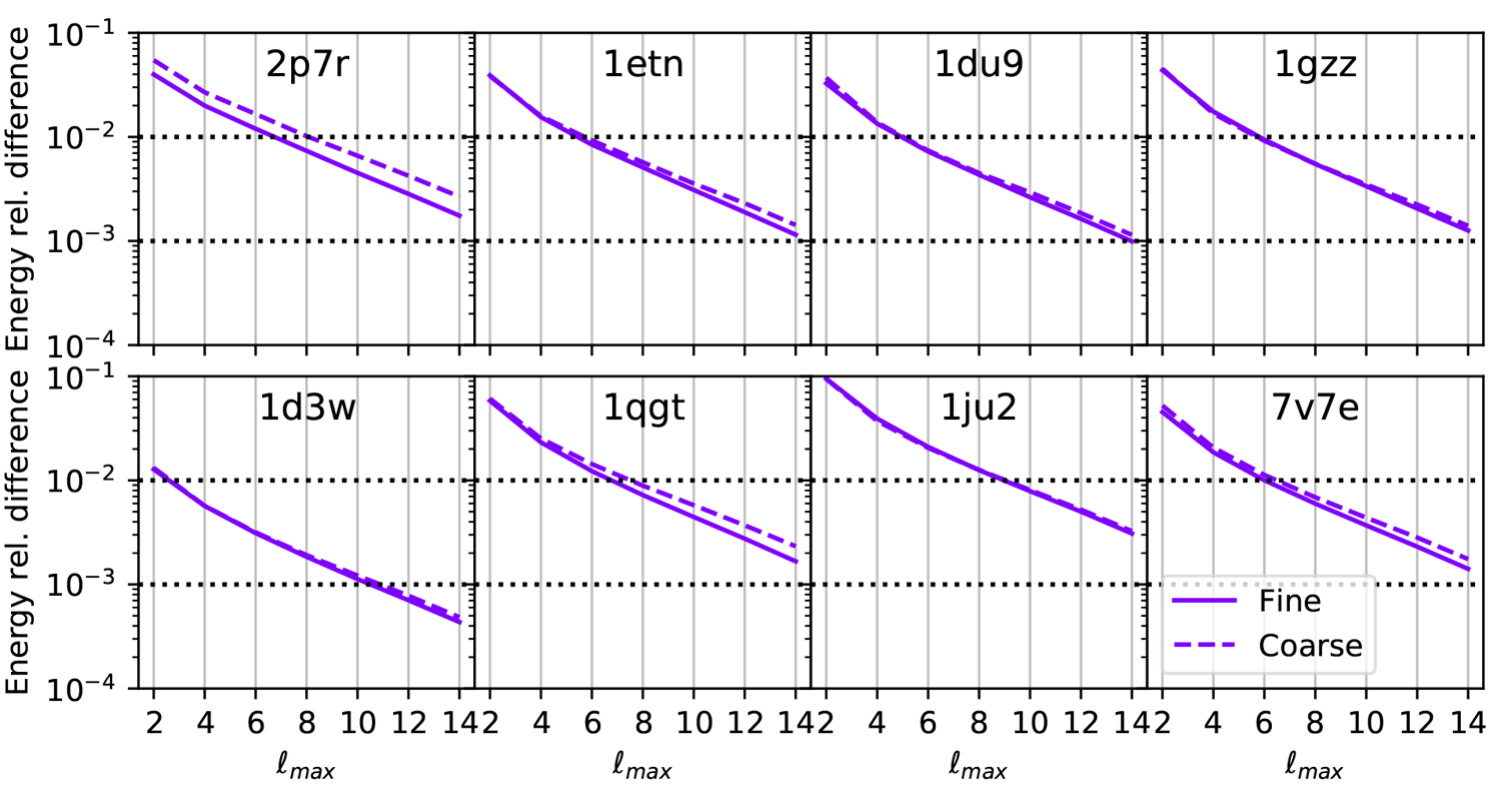
Relative differences on the energies for various structures and maximum angular momenta of the spherical harmonics. The reference values are the energies obtained using a coarse- and fine-grained model for . The relative differences of 1% and 1‰ are highlighted using horizontal dotted lines.

The relative differences are reported in Figure 5 for the various systems and various maximum angular momenta. For all the structures we observe the same trend, the difference exponentially decreases with an increasing . The results obtained with a coarse-grained model also follow the fine-grained ones, with an exception for the smallest system, where the two convergence profiles are slightly different. In general, an accuracy of ∼1% is obtained with a value of ; higher accuracies of ∼1‰ require a .

#### Effect of the Discretization on the Relative Stability of Conformers

To further assess the quality of the discretization, and to validate the parameters proposed in the previous section, we performed an analysis of the relative stability of a set of conformers. The structures were taken from the PDB file 1du9 (details are given in Table 1), which contains the structures of 25 conformers (only the first was used in the previous section). The conformers have been labeled with the letters from “a” to “y” following the same order given in the PDB file; the conformer presenting the lowest solvation energy is the “b” and is used as a reference for computing the energy differences. In these calculations we set  to the values 6 and 12 and a reference value of 20, *N*_grid_ = 2030 and *p*_max_ = 4 for the fine-grain and  for the coarse-grain.

**Figure 6:**
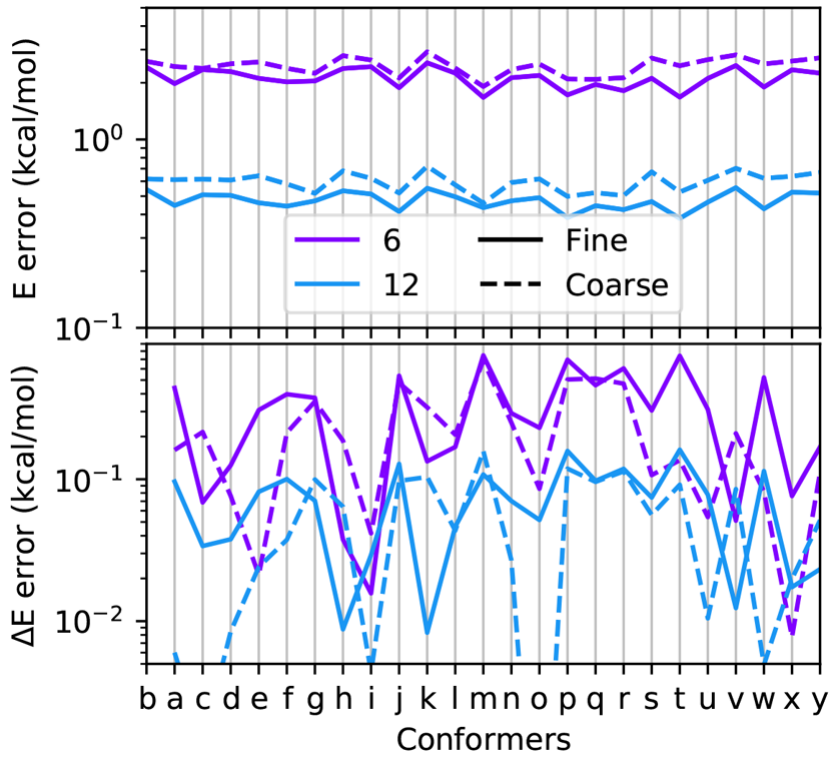
Top: absolute error on the solvation energy of the 25 conformers, Bottom: absolute error on the relative energies of the conformers with respect to the energy of conformer “b”. In both cases, the reference values are computed with .

Figure 6 reports the absolute error in kcal/mol of the energies of the conformers and the absolute error on the energy differences with respect to conformer “b”. As it can be observed, the error on the relative energies is roughly 1 order of magnitude smaller, this is because the discretization error is systematic in the positive direction (as it can be seen from Figure 5), so when energy differences are computed, there is an error cancellation that allows to use lower values of . For the analyzed system, using  already is sufficient to obtain errors on the solvation Δ*E*, which are below 1 kcal/mol. The same trend is observed for both the fine- and coarse-grained calculations. In a fine-grained calculation the energetic order of the conformers is stable after , whereas in a coarse-grained calculation it is stable after .

#### Comparison of the Fine- and Coarse-Grained Results

Once the optimal parameters for achieving an accuracy of ∼1% were determined, we ran a series of benchmarks on structures with numbers of atoms spanning all the orders of magnitude from 10^2^ to 10^6^. These calculations were obtained using , *N*_grid_ = 110 (which we tested to be enough to integrate the spherical harmonics with ), and in the fine-grained case, *p*_max_ = 4, whereas in the coarse-grained case we used *p*_max_ = 6. The calculation on the largest system was only possible with the coarse-grained model due to a high memory usage using the fine-grained model. We note here that a consistent memory usage (∼80 GB for the largest system) comes also from the allocations made by Tinker.

**Figure 7:**
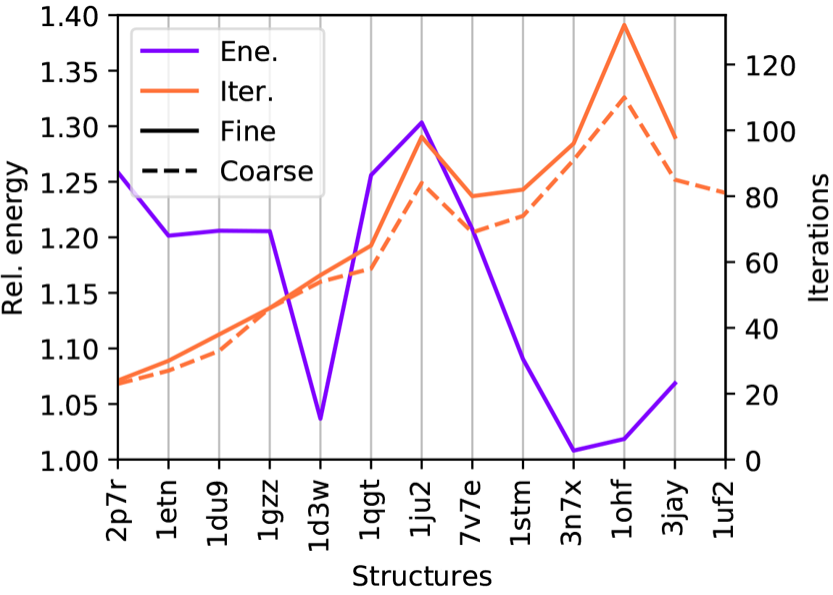
Purple: energy obtained with a coarse-grained calculation divided by the correspondent fine-grain energy for the various structure. Orange: number of iterations required by the Jacobi/DIIS solver for a fine- and a coarse-grained calculation, respectively, reported using solid and dashed lines.

Figure 7 reports the ratio between the coarse- and fine-grained energies, as well as the number of iterations in the two cases. The effect of the coarse-graining on the energy for a SAS cavity is a small increase in its value: all the coarse-grained energies are between 1× and 1.3× larger than the correspondent fine-grained ones. This can be rationalized by the fact that the charges of the hydrogens are closer to the cavity boundary, and hence, the polarization effects are stronger. We remark here that a sizable difference in the electrostatic contribution to the solvation energies between calculations performed with different molecular surfaces is to be expected, the only important thing being that the computed values are of the same order of magnitude. What we report are, in fact, not solvation-free energies, but only one of the contributions. Computing free energies would require one to parametrize the nonelectrostatic contributions and add them to the computed electrostatic energies. Such a parametrization is currently being investigated for both cavities and will be the object of a future communication. Another important consequence of the coarse-graining is that, simplifying the molecular cavity affects the number of iterations required for the solution of the linear system: their number is significantly lowered and, thus, the efficiency of the method is increased.

**Figure 8:**
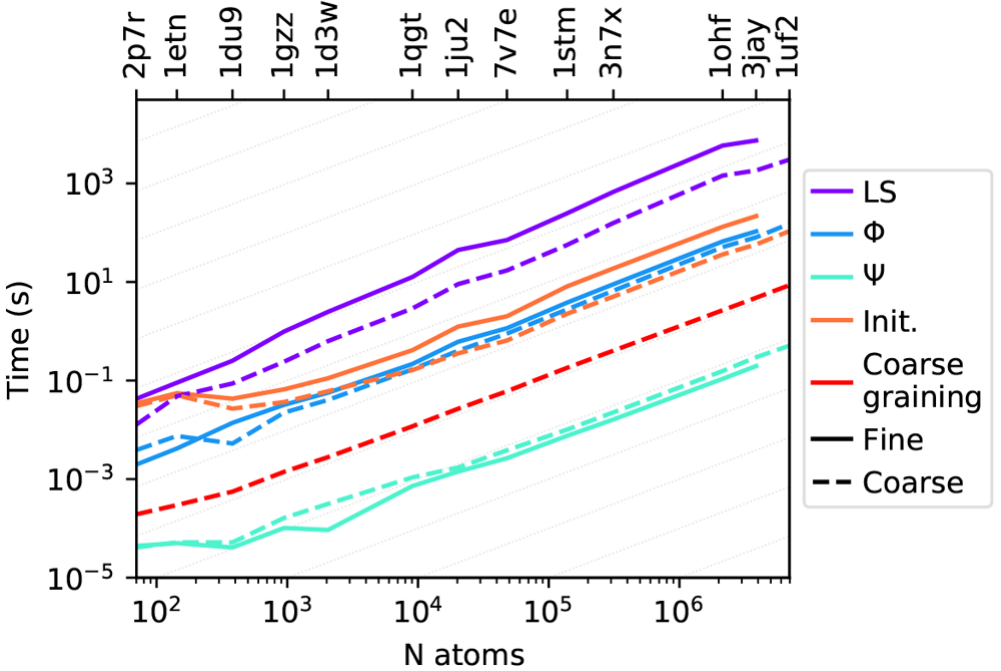
Time required to complete all the steps of a ddCOSMO calculation in a fine- and in a coarse-grained case, respectively, reported using solid and dashed lines. The labels refer to solving the linear system (LS), computing the electric potential (Φ), computing the representation of the solute density (Ψ), initialization (Init.), and coarse-graining. The light gray guidelines show the linear scaling regime.

Figure 8 reports the timings for all the steps required by a ddCOSMO calculation: solving the linear system, assembling the RHSs **Φ** and **Ψ**, plus the initialization and the time required for the coarse-graining. Table 2, on the other hand, reports the total time required by the ddX library, sum of all the steps reported in the plot. All the steps show that the computational cost scales linearly with the size of the system, with a small exception for the cost of the linear system, which is slightly more than linear scaling: despite the cost for a single iteration being linear, for the systems below 10^4^ atoms, we observe that the number of iterations increases with the size of the system (Figure 7). As expected, the most expensive step is solving the linear system, all the other steps are at least 1 order of magnitude faster, with the computation of **Ψ** and the coarse-graining being almost negligible on the total.

**Table 2:** Total Time Required by the ddX Library in Fine- and Coarse-Grained ddCOSMO Calculations

	fine-grain			coarse-grain		
system	h	m	s	h	m	s
2p7r	0	0	0.08	0	0	0.05
1etn	0	0	0.15	0	0	0.07
1du9	0	0	0.31	0	0	0.15
1gzz	0	0	1.08	0	0	0.28
1d3w	0	0	2.65	0	0	0.75
1qgt	0	0	13.25	0	0	3.30
1ju2	0	0	46.39	0	0	10.05
7v7e	0	1	14.15	0	0	18.77
1stm	0	4	19.90	0	1	3.07
3n7x	0	11	41.72	0	2	50.66
1ohf	1	40	59.34	0	23	48.18
3jay	2	9	20.22	0	32	42.42
1uf2				0	56	9.21

**Figure 9:**
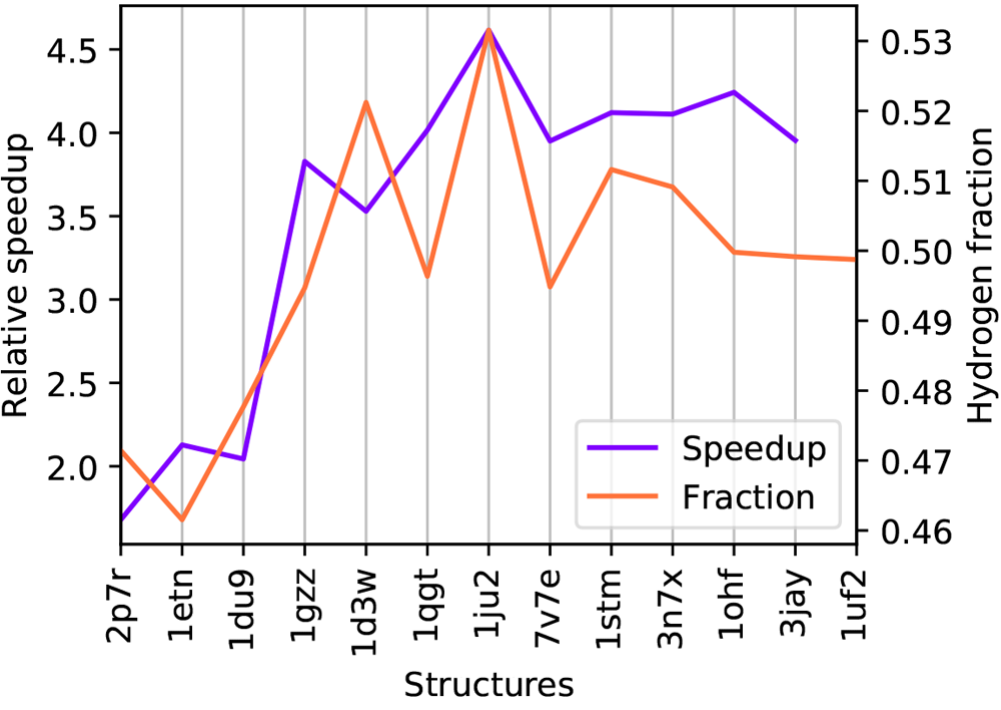
Purple: total time required by a fine-grained calculation divided by the total time required by a coarse-grained calculation for the various structures. Orange: fraction of hydrogens on the total number of atoms for the various structures.

Switching to a coarse-grained description results in a significantly lower time for the three most expensive steps, more about this is reported in Figure 9. For all the investigated structures, a coarse-grained calculation is between 1.7 and 4.6 times faster than a fine-grained calculation. As expected, the speedup shows a correlation to the fraction of hydrogens which are removed in the coarse-graining. However, the reduction in time associated with the coarse-graining is not exactly linear in the number of removed atoms: this is due to the fact that not only the number of spheres decreases, but also the average number of neighbors and the number of iterations is lower in the coarse-grained case.

## Conclusions

In this paper we presented a Tinker–ddX interface that can be used to compute the electrostatic contribution to the solvation energy for systems described using non polarizable force fields. The interface was also adapted to implement the united atom coarse-graining strategy, making it possible to test the coarse-grained ddCOSMO against the regular fine-grained ddCOSMO on a large variety of systems.

In our tests, we first identified discretization parameters that allow for an accuracy on the solvation energies of ∼1% and we then computed the solvation energies of systems with a number of atoms spanning all the order of magnitude from 10 to 10^6^. From the results, we found that the coarse-graining strategy applied to ddCOSMO is very effective at reducing its computational cost by both reducing the cost associated with a matrix-vector multiplication, and reducing the number of iterations required to converge the solution. For the largest systems, applying the coarse-grain results in a significant 4-fold increase in efficiency. At the same time, the discretization error is unchanged, and the energies obtained with coarse-grained calculations are comparable with those obtained with fine-grained calculations. Overall, we showed that ddCOSMO is a promising numerical strategy to compute the solvation energy for systems of any size, ranging from small systems as the ones used in high-accuracy quantum-mechanical applications, to very large ones, bridging thus the continuum solvation gap between quantum chemistry and biophysics.

A mandatory future development to make this strategy viable for practical applications is the parametrization of the nonelectrostatic contributions to the solvation free energy, for both fine- and coarse-grained cavities. A further natural continuation of this work is extending the support to more advanced force fields, such as the AMOEBA force field,^62−65^ which contains higher order multipoles and induced dipoles, and implementing the code required for the computation of the forces, both for fine- and coarse-grained calculations.

Finally, different, more aggressive, coarse-graining strategies could be devised and tested as well, which would allow applying ddCOSMO to systems even larger than those presented in this contribution.
